# Luminescent Composite Carbon/SiO_2_ Structures: Synthesis and Applications

**DOI:** 10.3390/bios12060392

**Published:** 2022-06-06

**Authors:** Yuliya A. Podkolodnaya, Alina A. Kokorina, Tatiana S. Ponomaryova, Olga A. Goryacheva, Daniil D. Drozd, Mikhail S. Khitrov, Lingting Huang, Zhichao Yu, Dianping Tang, Irina Yu. Goryacheva

**Affiliations:** 1 Department of Inorganic Chemistry, Chemical Institute, Saratov State University, Astrakhanskaya Street 83, 410012 Saratov, Russia; podkolodnaya00@mail.ru (Y.A.P.); tatyanka.ponomareva.97@mail.ru (T.S.P.); olga.goryacheva.93@mail.ru (O.A.G.); drozddd@sgu.ru (D.D.D.); mikkhitrov@yandex.ru (M.S.K.); goryachevaiy@mail.ru (I.Y.G.); 2 Key Laboratory for Analytical Science of Food Safety and Biology, Department of Chemistry, Fuzhou University, Fuzhou 350108, China; 201310011@fzu.edu.cn (L.H.); 201320082@fzu.edu.cn (Z.Y.); dianping.tang@fzu.edu.cn (D.T.)

**Keywords:** luminescent composite particles, carbon nanostructures, luminescent carbon-based nanomaterials, silica nanoparticles, luminescence

## Abstract

Luminescent carbon nanostructures (CNSs) have attracted great interest from the scientific community due to their photoluminescent properties, structural features, low toxicity, and a great variety of possible applications. Unfortunately, a few problems hinder their further development. These include the difficulties of separating a mixture of nanostructures after synthesis and the dependence of their properties on the environment and the aggregate state. The application of a silica matrix to obtain luminescent composite particles minimizes these problems and improves optical properties, reduces photoluminescence quenching, and leads to wider applications. We describe two methods for the formation of silica composites containing CNSs: inclusion of CNSs into silica particles and their grafting onto the silica surface. Moreover, we present approaches to the synthesis of multifunctional particles. They combine the unique properties of silica and fluorescent CNSs, as well as magnetic, photosensitizing, and luminescent properties via the combination of functional nanoparticles such as iron oxide nanoparticles, titanium dioxide nanoparticles, quantum dots (QDs), and gold nanoclusters (AuNCs). Lastly, we discuss the advantages and challenges of these structures and their applications. The novelty of this review involves the detailed description of the approaches for the silica application as a matrix for the CNSs. This will support researchers in solving fundamental and applied problems of this type of carbon-based nanoobjects.

## 1. Introduction

Luminescent CNSs are colloidal quasi-spherical nanoparticles consisting of amorphous and/or nanocrystalline carbon structure, oxygen/nitrogen groups, and bright and tunable emission [1,2].

Currently, CNSs are relatively new luminescent labels for biological and analytical applications [3]. Their optical properties are comparable or even superior to luminescent semiconductor QDs [3,4], upconversion nanoparticles [5], and organic dyes [6]. Moreover, CNSs have excellent solubility in water, high resistance to photobleaching, absence of toxic components, and biocompatibility [1,3,4,5]. CNSs can be synthesized from a wide range of start materials without expensive and time-consuming steps [7].

The structure of CNSs has not been fully explored. There are several theories about the structure of CNSs. One of them describes the structure of CNSs as amorphous carbon [8], another reports the crystal structure [9], and a third assumes the presence of a mixture of crystalline and amorphous components [10]. Moreover, there is a disproportion across the structural information provided by various methods, such as X-ray diffraction, high-resolution transmission electron microscopy (HRTEM), and Raman spectroscopy [3].

The nanostructures described in the literature can be called carbon dots [5,6,11], carbon nanoparticles [12,13], carbon quantum dots [14,15], etc. A uniform terminology has not yet been formed. In this review, we use the term “carbon nanostructure” due to their understudied structure and morphology.

Various start materials, synthetic approaches, and conditions allow obtaining CNSs with a wide luminescence range. Despite thousands of studies, there are several scientific open questions about the relationship between structure and emission, optical behavior, and the nature of electronic states [3,4].

Different synthetic methods lead to mixtures of CNSs with various properties. The actual purification and fractionation techniques are mostly multistage, costly, or low-scale productive [12]. The properties of CNSs can depend on the environment, which limits their applications [16]. Moreover, solid-state CNSs usually have low luminescence due to the quenching effect caused by the aggregation of nanoparticles [17].

Some of these problems can be solved by using a “fixation” matrix for CNSs. There is continuous exploration for a suitable matrix for composite formation with CNSs to preserve and even improve their properties. The literature reports various examples of matrices such as silica [18,19,20,21,22,23], polymers [24,25,26], starch [27], zeolites [28,29,30], potassium aluminum sulfate [31], trisodium citrate [32], and sodium chloride crystals [33]. Currently, silica is a powerful and effective matrix thanks to its optical transparency [34,35], biocompatibility [34], low toxicity [34,36], tunable dielectric properties [37], and potential for size and surface customization [34,36].

**Figure 1:**
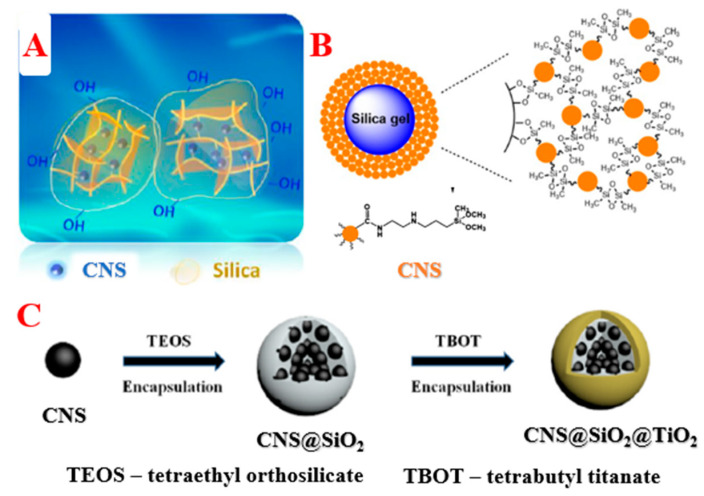
Schematic illustration of the composite formation: inclusion of CNSs into the silica matrix (**A**) [38], grafting of CNSs onto the silica surface (**B**) [22], and synthesis of bifunctional complexes (**C**) [39]. Adapted with permission from [38], ACS 2019, [22], Springer 2017, and [39], Elsevier 2020.

In this review, we summarize and provide a critical analysis of the synthesis of luminescent composite particles based on CNSs and silica spheres. We demonstrate two dominant methods for obtaining composites: (i) inclusion of CNSs into silica particles (Figure 1A) and (ii) grafting of CNSs onto the silica surface (Figure 1B). Additionally, we demonstrate the obtainment of bifunctional composites consisting of CNSs/silica and fluorescent, magnetic, or photosensitizing particles (Figure 1C). We analyze the advantages and disadvantages of these approaches and complexes, as well as describe their properties and the possibilities of their application (Table 1). 

## 2. Carbon Nanostructures: Features, Structure, and Properties

An unknown highly luminescent material was found in 2004 during the purification and separation of single-walled carbon nanotubes (SWNTs) synthesized from arc-discharge soot [42]. The separation of SWNTs from the species of arc soot was achieved by electrophoresis in agarose gel. The soot was previously oxidized by nitric acid and then extracted with basic water (pH 8.4). After the gel-electrophoretic separation, slow-moving bands corresponded with SWNTs, while fast-moving bands contained the highly luminescent carbon material. This material was separated into several fractions with different emissions in the green-blue, yellow, and orange regions. Since then, similar materials have been called carbon dots, carbon nanoparticles, etc., without a common terminology. In this review, we generally refer to these types of PL materials as CNSs. It is universally acknowledged that the above-described study [42] discovered CNSs for the first time [1,2,7].

These types of nanoparticles have been synthesized and studied by numerous scientific groups to determine the causes of their photophysical properties, improve their synthesis, and identify applications [3,11]. CNSs are nanoparticles with a unit size of nanometers, usually consisting of carbon, hydrogen, oxygen, nitrogen, or sulfur [43]. Their most important property is bright luminescence in the range from blue to near-infrared [44].

**Figure 2:**
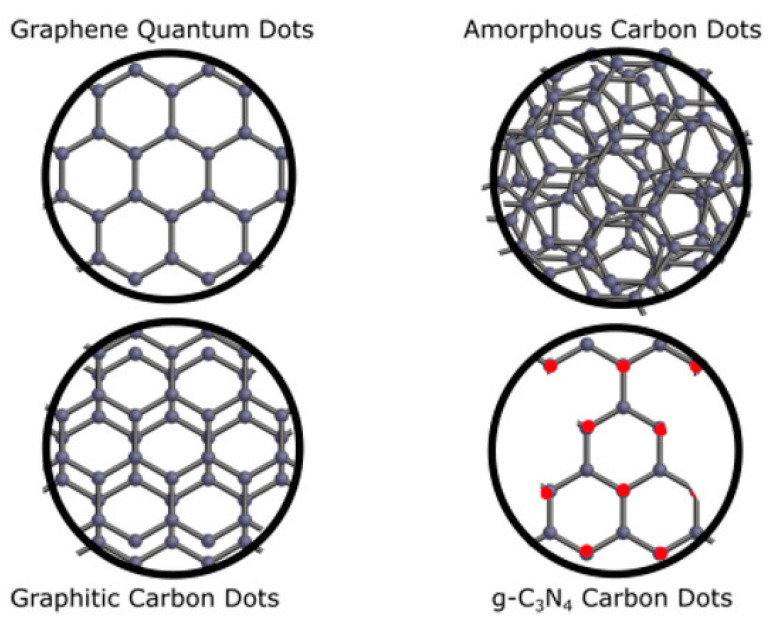
Schematic illustration of reported four types of CNSs. Black and red dots represent carbon and nitrogen atoms, respectively. Reprinted from ref. [4].

Luminescent CNSs are a large class of carbon-based nanosystems of various structural types. Messina’s research group classified CNSs according to their differences in structure and morphology. They proposed four groups of CNSs: graphene quantum dots, graphitic carbon dots, g-C_3_N_4_ carbon dots, and amorphous carbon dots (Figure 2) [4].

Graphitic carbon dots are spherical and the most popular type of nanostructures in the scientific literature [45,46,47,48,49]. However, a detailed study of their structural features by HRTEM was obtained in a few studies [47,50,51]. Their structure consists of several layers of *sp^2^*-hybridized carbon with a diameter of units of nanometers. Graphene quantum dots have a non-spherical structure with 1–3 graphene layers [52,53]. The optical properties of these CNSs are very similar. In fact, their assignment to different types is a subject of discussion.

The third type of CNSs is C_3_N_4_ carbon dots. Some research groups [54,55,56] reported on carbon nitride structures with a graphitic- or β-crystalline arrangement (g-C_3_N_4_ or β-C_3_N_4_). CNSs of the g-C_3_N_4_ type have a layered structure similar to graphite with *sp^2^*-hybridized carbon and nitrogen atoms. In contrast, β-C_3_N_4_ nanostructures have a hexagonal network of *sp^3^*-hybridized carbon atoms connected with *sp^2^*-hybridized nitrogen atoms.

Many studies have described a fourth type of CNSs—amorphous carbon dots [8,57,58]. They are a mixture of differently hybridized carbon (*sp^2^* and *sp^3^*) in various proportions without a specific core. However, they also exhibit bright luminescence.

Moreover, Zhu and et al. [59] presented polymer dots consisting of aggregated or crosslinked polymers derived from linear polymers or monomers.

Several research groups have presented fluorophore-connected CNSs. These fluorophores are formed during the synthesis of CNSs and can be free or connected to the surface of nanostructures [60,61]. The luminescence of these CNSs has been specified.

Similar sizes (≤10 nm) and surface functional layers characterize all described types of CNSs. These features determine the optical properties of the CNSs [3,4]. CNSs have absorbance in the far-ultraviolet (UV), visible, and near-infrared regions. Usually, intense absorption bands of CNSs have been observed in the range of 190 to 500 nm, corresponding to π→π* or n→π* transitions [4]. It is assumed that these absorption bands are determined by electronic transitions of functional groups located on the surface, as well as defects of the carbon core [4]. The position and intensity of optical bands depend on the temperature, pH, solvent, etc. [4,11].

The most attractive optical characteristic of CNSs is photoluminescence (PL). The spectral range and intensity of the PL bands of CNSs depend on their composition, type, and density of functional groups, and size, as well as energy of the excitation and properties of the environment [3,62]. Typically, CNSs have luminescence bands in the range of 350–700 nm [62]. A distinctive feature of the PL of CNSs is a strong dependence of emission on excitation; an increase in the excitation wavelength leads to a redshift of the spectrum band [62,63]. Researchers have correlated this fact with the presence of different emitting centers [64] or the emission of different chromophore groups in the CNS structure [65]. It should be noted that the optical properties of CNSs depend on the initial components and the synthetic technology [3]. In some cases, it is possible to improve the optical properties by varying the temperature, synthetic time, solvents, concentration, and ratio of starting substances [63]. However, there is no fundamental correlation among the synthetic method, structure, and properties of CNSs. The application of the matrix allows obtaining a better understanding of CNS structure and increases their applicability.

## 3. Silica Matrix: Synthetic Methods and Properties

Silica structures are often used as a matrix for nanoparticles [35,66,67,68,69] due to their thermodynamic and chemical stability in various chemical environments, low toxicity, biodegradability, optical transparency, and high surface area [34,69,70]. The presence of hydroxyl groups on the silica surface leads to high hydrophilicity and contributes to further functionalization [34].

Silica particles can mainly be synthesized by the Stober or the reverse microemulsion methods [36]. The Stober method can be applied to produce colloidal silica particles ranging from 200 to 2000 nm. This method is based on the hydrolysis and condensation of silanes in an aqueous alcohol solution in the presence of an ammonium hydroxide catalyst. The shape and size of the formed silica particles depend on the synthetic parameters: the concentrations of silane, ammonium hydroxide, water, and alcohol, their ratios, and the rate of their interaction [34,70,71]. However, researchers have described difficulties when synthesizing particles with target dimensions and shapes using the Stober approach because of the unlimited hydrolysis of silanes [71]. The reverse microemulsion method can solve these issues.

The reverse microemulsion method involves the formation of a thermodynamically stable dispersion of water in a nonpolar solvent stabilized by surfactant molecules called reverse micelles. Reverse micelles serve as nanoreactors for the hydrolysis and condensation of silanes to form silica nanoparticles. This method allows obtaining a narrow particle size and shape distribution because of similar micelle reactor sizes. In this case, the size and shape of the resulting silica nanoparticles depend on several factors: the nature of the organic solvent and surfactant, the molar ratio of reverse micelle components, the concentration of ammonium hydroxide, the synthesis time, and the amount of tetraethylorthosilicate (TEOS) and/or other organosilanes for the surface modification of nanoparticles [36,69,72].

## 4. Formation of Composite Carbon/SiO_2_

The following problems limit the application of CNSs:

I. Obtainment of a polydisperse product;
II. The dependence of their optical properties and stability on the chemical environment;
III. PL dependence on properties of the microenvironment and the quenching of luminescence in the lyophilized samples;
IV. Nonuniform distribution of surface functional groups;
V. Weak PL intensity.

The above-listed problems can be solved by the application of a silica matrix. CNSs can be associated with silica via two approaches: inclusion of CNSs into the matrix or grafting of CNSs onto the silica surface. Moreover, these approaches can be applied to the synthesis of bifunctional complexes.

### 4.1. Inclusion of CNSs into the Silica Matrix

**Figure 3:**
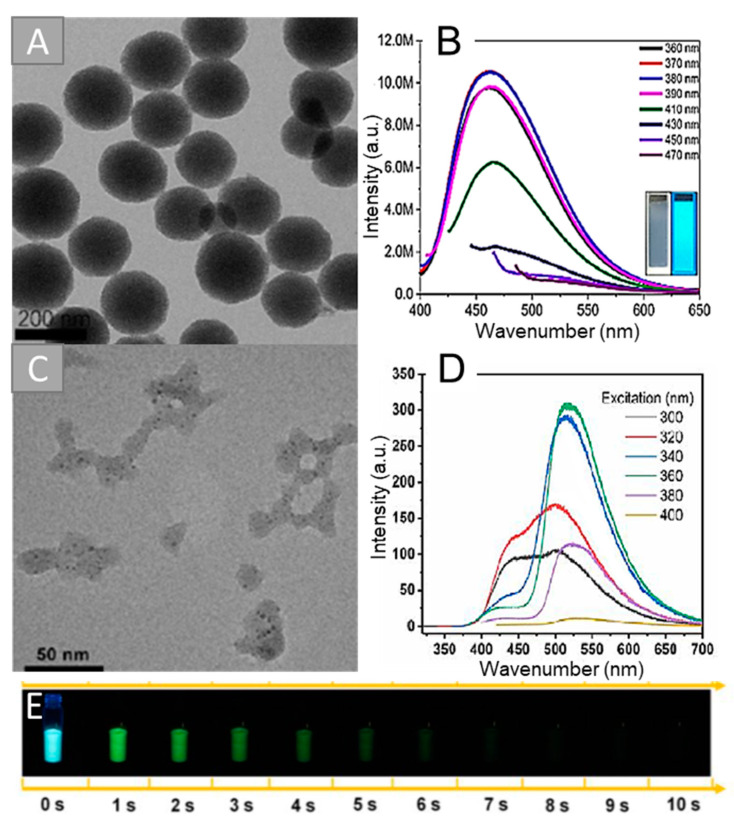
TEM images and emission spectra of the CNSs/SiO_2_ composite obtained by reverse microemulsion method after separation (**A**,**B**) [18] and synthesized by Stober method (**C**,**D**) [38]. Images of solid-state composite after 365 nm irradiation from 0 to 10 s (**E**) [38]. Adapted with permission from [18], ACS 2019, and [38], American Chemical Society 2019.

The inclusion of CNSs into silica spheres allows solving the above-stated problems. Thus, Xu’s group [18] synthesized luminescent composite nanoparticles via simple co-hydrolysis of CNSs with TEOS. The authors synthesized amino-modified CNSs via pyrolysis of citric acid (CA) and *N*-(β-aminoethyl)-γ-aminopropylmethyldimethoxysilane (AEAPTMS) at 240 °C for 5 min under nitrogen atmosphere. The hydrolysis of TEOS and the obtained CNSs was carried out by the reverse microemulsion method. However, the authors highlighted the polydispersity of the luminescent composite nanoparticles. They applied differential centrifugation to obtain uniform-sized nanoparticles. Figure 3A shows a transmission electron microscope (TEM) image of the composites after separation. The PL maximum of the obtained particles was in a blue region at 460 nm and depended on the excitation wavelength (Figure 3B). The proposed approach allowed increasing the composite PL quantum yield (QY) by 9% compared to the initial CNSs (56% and 47%, respectively). The authors described the homogeneous distribution of CNSs in the SiO_2_ matrix, as well as the good reproducibility achieved by this approach. This composite was used in an ultrasensitive method for the detection of the thrombocytopenia syndrome virus (SFTSV). The synthesized composite nanoparticles were used as labels for immunochemical analysis. The composite was conjugated with an anti-SFTSV monoclonal antibody for the registration of the optical signal. The detection limit of SFTSV was 10 pg/mL. The sensitivity of the developed assay was two orders of magnitude higher than that of the colloidal gold-based test method. The authors claimed that this method can be used for other viruses, protein biomarkers, nucleic acids, and bacteria in clinical diagnostics.

The Zhao group [19] used a similar technique for the synthesis of composite nanoparticles at 234 °C for 5 min. The obtained particles had a spherical morphology and sizes of 84 to 190 nm. These composite nanoparticles were successfully used for detecting latent fingermarks. The porous structure of the composite increased the adhesion of the nanoparticle to fingermarks. Moreover, these nanocomposites were effective and sensitive for both fresh and aged fingermarks. The composites were sensitive for the detection of latent fingermarks in a range of substrates such as glass, aluminum foil, plastic bags, drug packing, and leather (the use of CNSs to improve the visualization of hidden fingerprints is only applicable to a smooth nonporous surface). The optical signal was obtained at 415 nm irradiation. The luminescence of the found fingermarks was bright and gave a fair contrast, which suggests that the composite selectively targets the latent fingermarks.

Song’s research group [17] obtained luminescent composite nanoparticles with multicolor emission via reverse-phase microemulsion. Firstly, the researchers synthesized multicolored CNSs using the one-step hydrothermal (HT) method from CA and urea. The authors varied the ratio of the initial components, reaction time (6/10 h), synthetic temperature (160/180 °C), and solvents (water, ethyl alcohol, and dimethylformamide (DMF)). The PL maxima of the CNSs were in the regions from 450 to 650 nm. The reagent ratio allowed achieving multicolor luminescence, and the HT temperature and time were adjusted to obtain more intensive optical properties. Secondly, a mixture of CNSs, TEOS, and 3-aminopropyltriethoxysilane (APTES) was added to the reverse-phase microemulsion. This synthetic method is effective for preventing CNS quenching caused by the aggregation of nanoparticles. The range of sizes from 18 to 159 nm of the obtained composite particles depended on the TEOS concentrations. The authors described the production of multicolor light-emitting diodes (LED) with the developed luminescent composite nanoparticles. A composite-based LED was successfully fabricated by varying the content ratio of blue-, green-, and red-emitting CNS/SiO_2_ with a Commission Internationale de L’Eclairage (CIE) of (0.3497, 0.3045) and color rendering index (CRI) of 85.2.

Li’s scientific group [38] used the Stober method for the synthesis of composite nanoparticles with CNSs from polyacrylic acid and ethylenediamine (EDA), obtained using the HT method at 200 °C for 8 h. For the composite synthesis, TEOS and CNSs were mixed in the presence of ammonia and heated at 100 °C with vigorous stirring. This method led to the covalent bonding of the CNSs to the matrix. Figure 3C shows HRTEM images of the obtained composites. CNSs were located inside amorphous silica structures. The obtained composites had a nonuniformity of size and shape. CNSs did not have phosphorescent properties, while the composites demonstrated phosphorescence at 520 nm (Figure 3D). The phosphorescence lifetime of the obtained composites was 1.64 s (Figure 3E). The silica matrix was a protective shield preventing quenching and enhancing solubility in water. The authors reported successful phosphorescence imaging of CNS/SiO_2_, both in vivo and in vitro, highlighting the advantage of long-lived phosphorescence in bioimaging by eliminating the autofluorescence interference, especially under short-wavelength excitation. The biocompatibility of CNSs@SiO_2_ composites was demonstrated on the mouse breast carcinoma EM-6 cell line. The MTT colorimetric assay verified the very low cytotoxicity of composites up to 150 μg/mL.

Thus, the inclusion of CNSs in the silica matrix increases the PL signal because the PL intensity of several CNSs is higher than the intensity of a single one. Moreover, the application of the matrix can reduce the influence of the chemical environment on the optical properties. Silica spheres allow avoiding the PL quenching associated with the aggregation of CNSs in the solid state. However, both reverse microemulsion and Stober methods generally do not provide a synthesis of monodisperse structures. The synthesis of luminescent composites, as a rule, consists of several stages and is quite time-consuming. Many researchers have used differential centrifugation as an additional step to obtain uniform size fractions of composites.

### 4.2. Grafting of CNSs onto the Silica Surface

Grafting is also a very convenient method since the PL properties of composite nanoparticles can be controlled by the concentration of CNSs during the synthesis. Moreover, this method allows introducing specific functionality to the composite surface. The grafting process proceeds through the formation of covalent bonds and increases the stability of the obtained composites.

**Figure 4:**
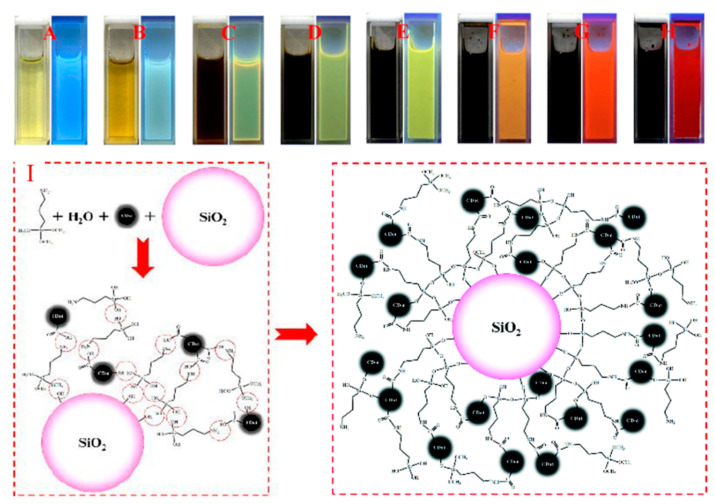
Images of CNSs solutions (**A**–**H**) with concentrations (6–240 mg/mL) under daily and UV light (λex. = 365 nm). Scheme of CNSs/SiO_2_ formation mechanism (**I**). Reprinted with permission from [40], Royal Society of Chemistry 2020.

Sun et al. [40] synthesized luminescent composite nanoparticles by grafting CNSs onto the surface of a silica matrix via an amide bond formation reaction. They used CA and urea (mass ratio = 1:2) in DMF via the solvothermal method in an autoclave at 160 °C for 8 h for CNS synthesis. An increase in the concentration of reagents (from 6 to 240 mg/mL) led to a gradual shift of the PL maximum to longer wavelengths (Figure 4A–H). The solvothermal reaction of citric acid and urea was demonstrated to be an available route to acquire full-color-emitting CNSs at controlled reactant concentrations in DMF. They mixed different concentrations of a solution of CNSs, 3-aminopropyltrimethoxysilane, and silica nanoparticles in DMF with stirring at room temperature for 3 h (Figure 4I). The reaction mixtures were centrifuged at 8000 rpm for 10 min, and the precipitated solids were solidified in a vacuum oven at 50 °C for 24 h. The size of the obtained composites was 25–40 nm. The use of a matrix prevented the quenching of the CNS luminescence in a solid state. The combined application of full-color-emitting CNS/SiO_2_ and InGaN chips with different peak wavelengths led to design flexible packaging schemes for white LEDs (WLEDs), obtaining a pure white light at the CIE coordinates of (0.33, 0.33) with CRI of 80.4 and a high color-rendering white light with CIE coordinates of (0.34, 0.36) and CRI of 97.4. This fact indicated the significant application potential of the SiO_2_/CNSs composite in the LED field.

The Thongsai group [23] used other methods for chemical grafting of CNSs onto the silica substrate. The authors synthesized CNSs from nylon-6 by pyrolysis at 250 °C for 6 h in the presence of sulfuric acid. The resulting product was HT treated with nitric acid at 200 °C for 6 h. The PL QY of the product was 2.53%. The obtained CNSs were grafted onto the silica surface primarily modified with APTES or 3-glycidyloxypropyltrimethoxysilane (GOPTMS). Grafting of CNSs was carried out via a carbodiimide reaction with EDC. The functionalized substrates were immersed and stirred in a solution with CNSs at room temperature for 24 h. The product was washed several times with deionized and sonicated to remove unreacted CNSs. The composites were dried with N_2_ and kept in a vacuum oven before use. The authors also described the preparation of composite nanoparticles via thermal annealing. A water solution of CNSs was added dropwise onto the purified silica substrate and subjected to thermal annealing in a vacuum chamber at a high temperature (160 °C). The data of the root-mean-square roughness and contact angle of the silica surface after grafting indicated successful CNS attachment. The authors noted that the functionalization of both APTES and GOPTMS was effective for introducing CNSs to the silica surface, but a uniform dense monolayer was not obtained. In addition, the two-stage methods required a long reaction time and several purification stages. Therefore, the one-stage method of thermal annealing is the most promising. Moreover, the successful grafting of CNSs onto APTES- and GOPTMS-terminated surfaces showed the presence of carboxyl and amino groups. The obtained composites were used for the determination of heavy-metal ions. The composite demonstrated selective detection and high adsorption of Cu^2+^ ions, suggesting practical applications as two-in-one sensors and adsorbents.

The Cai group [22] successfully grafted CNSs onto the matrix surface without linkers. The authors synthesized silanized CNSs via pyrolysis of CA in hot AEAPTMS at 240 °C for 5 min, which were grafted on the silica micro-sized particles. A mixture of silanized CNSs and silica microparticles was sonicated, heated to 110 °C, mechanically stirred for 24 h, and mixed until the formation of a homogeneous dispersion. After that, the resulting substance was washed sequentially with toluene and ethanol and dried at 60 °C in a vacuum oven. This method allowed obtaining a uniform distribution on the silica surface. The resulting luminescent composite particles had a large surface area and a variety of functional groups, high adsorption, and thermal and mechanical stability. These composites were used as a stationary phase in hydrophilic interaction chromatography for the separation of sulfonamides, flavones, amino acids, nucleosides, and bases. The composite stationary phase was packed into stainless-steel columns (150 mm × 4.6 mm i.d.) with carbon tetrachloride as the slurry solvent and acetonitrile as the mobile solvent. This work revealed a new way to enhance the chromatographic selectivity by CNSs, which increases the density of the interaction sites on the stationary phase.

The synthesized composite nanoparticles from CNSs and silica helped to better understand the structure of CNSs, to consider surface and interfacial phenomena with their participation (such as adsorption, energy, and charge transfer), and to apply CNSs in important applications (sensing, chromatography, LEDs). However, the chemical grafting of CNSs onto the silica surface is a complex procedure and requires appropriate chemical reactions. The authors note the difficulties in obtaining a monolayer of CNSs on the surface of silica. The resulting composite requires several stages of purification to separate uncombined CNSs.

### 4.3. Formation of Bifunctional Structures

Composite nanoparticles can be used to obtain bifunctional complexes with a simultaneous attachment of structures both inside and on the surface of silica. The application of a silica matrix allows solving the previously described problems, as well as combining the unique fluorescent, magnetic, and photosensitizing properties of particles of different nature.

Guan et al. [41] fabricated bifunctional composites from Fe_3_O_4_, SiO_2_, and CNSs. A single-stage microwave method was applied for CNS synthesis from urea and folic acid at 800 W for 8 min. Magnetic nanoparticles Fe_3_O_4_ were synthesized using the solvothermal method from iron chloride hexahydrate and anhydrous sodium acetate in a mixture of ethylene glycol and diethylene glycol at 200 °C for 12 h. Then, Fe_3_O_4_ particles were silanized using the classical Stober method with TEOS and coated with APTES for amino modification of the SiO_2_ surface. The carbodiimide method was used for grafting of CNSs onto the amino-modified Fe_3_O_4_/SiO_2_ surface with EDC and NHS linkers for functional group activation. The magnetic–fluorescent composite had a size of 155 nm and PL maximum at 455 nm. The PL intensity of the composite was lower than that for CNSs. This fact can be explained by the possible interaction of functional groups on the surface of the CNSs with iron ions. The resulting composites had a magnetic saturation intensity of 31.2 emu/g. In vitro experiments for Fe_3_O_4_/SiO_2_/CNSs to load and release gambogic acid in PBS (pH = 7.4 or 5.7) were provided. The cell uptake experiments were performed by incubating the cell line with bifunctional nanoparticles. Blue luminescence from CNSs in Fe_3_O_4_/SiO_2_/CNSs nanoparticles was observed near the nucleus, indicating that these nanoparticles penetrate the cells via endocytosis. The authors reported the successful application of the obtained composite in synergistic therapy, including the release of gambogic acid and magnetic targeting. The release of gambogic acid led to the inhibition of tumor cells, and their survival rate was less than 20% at a concentration of 100 μg/mL.

A similar synthetic procedure was applied by Xu’s research group [21]. They obtained luminescent composites via covalent linking of blue-emissive CNSs with the surface of silica nanoparticles containing red-emissive (λ_em_. = 658 nm) quantum dots. The QD-embedded silica nanoparticles were synthesized with TEOS hydrolysis and condensation using the Stober method, and their surface was modified with APTES. CNSs were obtained from CA and EDA using the microwave approach at 750 W for 10 min and contained carboxyl groups on their surface. These functional groups reacted with amino-modified silica nanoparticles. The size of the composites was ~50 nm. The obtained composite nanoparticles had PL maxima at 453 and 658 nm under the excitation of 350 nm. The obtained composites were used for the determination of mercury ions. The intensity of the PL maximum at 453 nm had a linear dependence on the concentration of mercury ions, while the luminescence at 658 nm was unchanged. The detection limit of mercury ions was 0.47 nM, corresponding to modern analytical standards.

**Figure 5:**
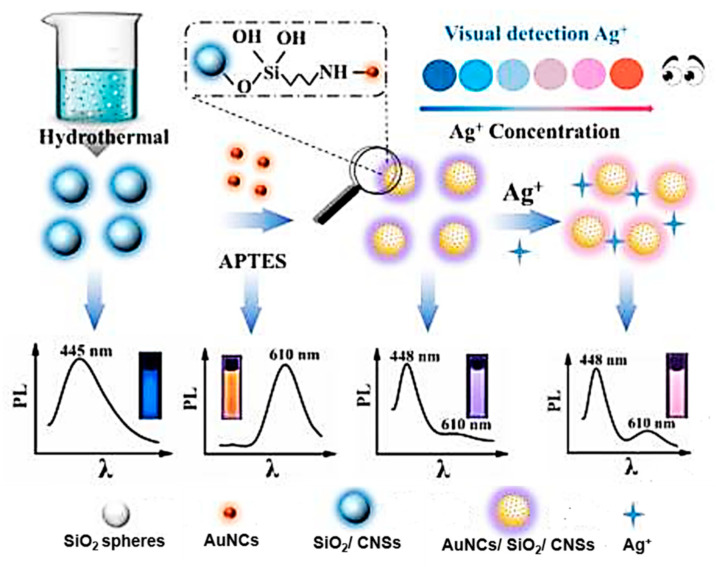
Schematic illustration of the composite synthesis and Ag^+^ detection. Reprinted with permission from [20], Elsevier 2021.

A different dual-emission ratiometric optical probe was developed by An’s group [20] (Figure 5). The authors HT treated CA, EDA, and silica spheres at 200 °C for 5 h. They suggested the simultaneous formation of CNSs and their fixation inside the silica nanoparticles during HT synthesis. The composite nanoparticles were covalently bonded with AuNCs. Nanoclusters were prepared thermally at 70 °C for 24 h from chloroauric acid and glutathione. AuNCs were pre-functionalized by APTES for covalent binding to the surface of composite particles. The size of the obtained composites was approximately 57 nm. The composites simultaneously displayed two emission maxima at 448 nm and 610 nm under a single excitation wavelength of 380 nm. The obtained composites were dispersed in buffer solutions with different pH values (from 2 to 12), while the PL intensity was the same, which indicates that the composites were stable in highly alkaline and highly acidic conditions. The developed composite was used to determine the level of silver ions; with an increase in their concentration, the PL in the region of 610 nm increased noticeably, while the PL in the region of 448 nm remained unchanged. The detection limit of Ag^+^ for this system was 1.6 nM. Thus, this method has advantages in Ag^+^ detection sensitivity compared to other analytical methods.

Bing et al. [39] also used CA and an amine-containing agent (urea) as precursors for the HT (150 °C, 5 h) production of CNSs. Composite CNSs/SiO_2_ particles were synthesized by mixing of CNSs with polyvinylpyrrolidone in ethanol and water followed by the addition of APTES and TEOS. The mixture was stirred for 3 h at 20 °C. The polyvinylpyrrolidone acted as a dispersant, structurant, and linker during the reaction. The synthesized composite particles CNSs/SiO_2_ were additionally wrapped with the photosensitizing agent of titanium dioxide as a shell. The CNSs inside the silica matrix had a multifunctional core for photothermal therapy and photothermal imaging. These composite nanoparticles had good biocompatibility and a uniform size of 150 nm. CNSs/SiO_2_ composites can be used for photothermal therapy and photothermal imaging due to the induction apoptosis in cancer cells. These structures could absorb long-wavelength light for the activation of photothermal effects and provide cancer therapy in deep tissues. Moreover, these composites responded to ultrasonic stimulation with a generation of the oxygen active forms for cancer sonodynamic therapy.

Thus, we demonstrated examples of silica application as a matrix for the synthesis of bifunctional composites. Researchers could control and adjust the concentration of the particles, as well as the size of the composites, at each stage of synthesis. However, the preparation of composites is a multistage process. Moreover, a few authors noted the quenching of PL due to the interaction of CNSs with the other components. These bifunctional systems can be used in sensing (detection of heavy-metal ions), drug delivery systems, bioimaging, and antitumor therapy.

## 5. Conclusions

CNSs have attractive characteristics such as unique optical properties, excellent biocompatibility and photostability, the possibility of surface functionalization, high colloidal stability, and low toxicity. To this day, the scientific community is looking for ways to solve fundamental and applied issues for the wider application of these nanoobjects. The issues include the unclear structure of CNSs and/or their luminescence mechanism, the dependence of properties on the environment and/or state of aggregation, and difficulties in their purification and separation. The application of a silica matrix for CNSs allows unifying the composite CNSs/SiO_2_ shape, size, and optical properties. Silica is a proper matrix due to its optical transparency, variety of functional groups for surface modifications, and controllable dimensions during synthesis.

We demonstrated examples of the inclusion of CNSs into the silica matrix or grafting onto its surface. Composite formation solves the problems associated with luminescence quenching in the solid state and purification of the product, as well as improves the luminescent properties via the emission of several CNSs. Moreover, silica and CNS composites led to a better understanding of the structure of CNSs, as well as surface and interfacial phenomena, such as adsorption, energy, and charge transfer. CNSs/SiO_2_ composites have improved brightness and chemical stability compared to CNSs.

Furthermore, the silica matrix makes it possible to obtain multifunctional nanocomposites due to the combination of several nanoparticles with different properties such as fluorescent, magnetic, or photosensitizing properties. This review reported examples of the successful application of composites in bioimaging, medicine, chemical, and immunoassays, in LED production, and as a stationary phase in hydrophilic interaction chromatography.

It is important to note that the approaches for composite synthesis require further improvement of the obtained structures with the necessary dimensions, while avoiding separation and purification steps. The development of effective and well-controlled strategies for the synthesis of these composites can lead to their wider application in medical, biological, analytical, or technical areas.

## Figures and Tables

**Table 1:** Examples of CNSs/SiO_2_ composites: synthesis, properties, and applications.

Synthesis CNS		Synthesis Composite		Composite		Application	References
Precursor	Method	Precursor	Method	Size, nm	Property		
CA, AEAPTMS	Pyrolysis	CNSs, TEOS	Hydrolysis by reverse microemulsion	~150	**PL**: **λ_ex_**. = 380 nm **λ_em_**. = 460 nm **QY** = 56%	Labels for immunochemical analysis	[18]
CA, AEAPTMS				84–190	**PL**: **λ_ex_**. = 380 nm **λ_em_**. = 460 nm	Detecting fingermarks	[19]
CA, urea	HT	CNSs, TEOS, APTES		18–159	**PL**: **λ_ex_**. = 365 nm **λ_em_**. = 450–650 nm	LEDs	[17]
Polyacrylic acid, EDA		CNSs, TEOS	Hydrolysis by Stober	-	**Phosphorescence**: **λ_em_**. = 520 nm	Labels in tissues	[38]
CA, urea	Solvothermic	CNSs, ARTMS, and silica nanoparticles	Chemical grafting	25–40	**PL**: **λ_ex_**. = 400 nm **λ_em_**. = 455–650 nm **QY** = 30–60%.	LEDs	[40]
1. Nylon 6, sulfuric acid 2. Obtained CNSs, nitric acid	1. Pyrolysis 2. Solvothermic	Two methods: (a) silica modified with APTES/GOPTMS, CNSs (b) Silica, CNSs	(a) Chemical grafting (b) Thermal annealing	-	**PL**: **λ_ex_**. = 360 nm **λ_em_**. = 460 nm **QY** = 2.5%.	Determination of ions	[23]
CA, AEAPTMS	Pyrolysis	CNSs, silica microsized particles	Sonication, mechanical mixing	>5000	-	Stationary-phase chromatography	[22]
Urea, folic acid	Microwave	Fe_3_O_4_, TEOS, APTES, CNSs	Hydrolysis by Stober and chemical grafting	155	**PL**: **λ_ex_**. = 370 nm **λ_em_**. = 455 nm **A magnetic saturation intensity**: 31.2 emu/g	Synergistic medicine	[41]
CA, EDA		CdTe, TEOS, APTES, CNSs		50	**PL**: **λ_ex_**. = 350 nm **λ_em_**. = 455 and 658 nm	Ratiometric optical labels	[21]
CA, EDA, silica spheres	HT	Silica spheres@CNSs, AuNCs, APTES	Chemical grafting	57	**PL**: **λ_ex_**. = 360 nm **λ_em_**. = 448 and 610 nm		[20]
CA, urea		CNSs, APTES, TEOS, TiO_2_	Hydrolysis by Stober	150	-	Photothermal and photodynamic therapy	[39]

## Abbreviations

AEAPTMS	*N*-(β-aminoethyl)-γ-aminopropylmethyldimethoxysilane
APTES	3-aminopropyltriethoxysilane
AuNC	gold nanocluster
CA	citric acid
CIE	Commission Internationale de L’Eclairage
CNS	carbon nanostructure
CRI	color rendering index
DMF	dimethylformamide
EDA	ethylenediamine
GOPTMS	3-glycidyloxypropyltrimethoxysilane
HRTEM	high-resolution transmission electron microscopy
HT	hydrothermal
LED	light-emitting diode
PL	photoluminescence
QD	quantum dot
QY	quantum yield
SFTSV	thrombocytopenia syndrome virus
SWNT	single-wall carbon nanotube
TEM	transmission electron microscopy
TEOS	tetraethyl orthosilicate
UV	ultraviolet
